# Supplementary material for: At the roots of Plant Awareness Disparity (PAD): Semantic processing and numerosity perception
Source: PLoS One. 2026 May 15;21(5):e0343890. doi: 10.1371/journal.pone.0343890 (PMC13178887; doi:10.1371/journal.pone.0343890)
Supplement: Attachment — Submitted filename: Response to Reviewers.pdf [file pone.0343890.s002.pdf]

**Editor comments:**

1. Please ensure that your manuscript meets PLOS ONE's style requirements, including those for file naming. The PLOS ONE style templates can be found at

https://journals.plos.org/plosone/s/file?id=wjVg/PLOSOne_formatting_sample_main_body.pdf
https://journals.plos.org/plosone/s/file?id=ba62/PLOSOne_formatting_sample_title_authors_affiliations.pdf

**R1.** We thank the Editor for raising this important issue. We have revised the manuscript to ensure full compliance with PLOS ONE style requirements. Please refer to the updated version of the manuscript.

2. PLOS requires an ORCID iD for the corresponding author in Editorial Manager on papers submitted after December 6th, 2016. Please ensure that you have an ORCID iD and that it is validated in Editorial Manager. To do this, go to 'Update my Information' (in the upper left-hand corner of the main menu), and click on the Fetch/Validate link next to the ORCID field. This will take you to the ORCID site and allow you to create a new iD or authenticate a pre-existing iD in Editorial Manager.

**R2.** We thank the Editor for this remark. The ORCID ID for the corresponding author has been added and validated in the Editorial Manager system.

3. We note that the grant information you provided in the 'Funding Information' and 'Financial Disclosure' sections do not match. When you resubmit, please ensure that you provide the correct grant numbers for the awards you received for your study in the 'Funding Information' section.

**R3.** We thank the Editor for highlighting this important point. We have revised the Funding Information section to ensure consistency and accuracy of the reported grant details.

**4.** Thank you for stating the following financial disclosure:
"This work was supported by the Department of General Psychology (DPG) BATT_BIRD24_01 - Plant Awareness Project: Numerosity, Training and Brain Stimulation – PRID (Interdisciplinary Department Projects) granted to L.B and S.G." Please state what role the funders took in the study. If the funders had no role, please state: "The funders had no role in study design, data collection and analysis, decision to publish, or preparation of the manuscript." If this statement is not correct you must amend it as needed. Please include this amended Role of Funder statement in your cover letter; we will change the online submission form on your behalf.

**R4.** We thank the Editor for highlighting this important issue. Please refer to the revised Financial disclosure: "This work was supported by the Department of General Psychology (DPG) BATT_BIRD24_01 - Plant Awareness Project: Numerosity, Training and Brain Stimulation – PRID (Interdisciplinary Department Projects) granted to L.B. The funders had no role in study design, data collection and analysis, decision to publish, or preparation of the manuscript."

**5.** Please note that funding information should not appear in any section or other areas of your manuscript. We will only publish funding information present in the Funding Statement section of the online submission form. Please remove any funding-related text from the manuscript.

**R5.** Funding information has been removed from the new version of the manuscript and is now reported exclusively in the Funding Statement section, as required. Please refer to the updated version of the manuscript.

**6.** Please note that your Data Availability Statement is currently missing the repository name and/or the DOI/accession number of each dataset OR a direct link to access each database. If your manuscript is accepted for publication, you will be asked to provide these details on a very short timeline. We therefore suggest that you provide this information now, though we will not hold up the peer review process if you are unable.

**R6.** We thank the Editor for raising this important issue. The dataset used in the present study is publicly available in the Open Science Framework (OSF) repository: https://osf.io/mzpr5/

The repository contains all the data required to replicate the analyses reported in this manuscript. Specifically, the following datasets were used:
The estimation dataset: https://osf.io/mzpr5/files/8f795
The comparison dataset: https://osf.io/mzpr5/files/u4dxs

**7**. We note that Figure 1 in your submission contain copyrighted images. All PLOS content is published under the Creative Commons Attribution License (CC BY 4.0), which means that the manuscript, images, and Supporting Information files will be freely available online, and any third party is permitted to access, download, copy, distribute, and use these materials in any way, even commercially, with proper attribution. For more information, see our copyright guidelines: http://journals.plos.org/plosone/s/licenses-and-copyright. We require you to either (1) present written permission from the copyright holder to publish these figures specifically under the CC BY 4.0 license, or (2) remove the figures from your submission: You may seek permission from the original copyright holder of Figure 1 to publish the content specifically under the CC BY 4.0 license. We recommend that you contact the original copyright holder with the Content Permission Form (http://journals.plos.org/plosone/s/file?id=7c09/content-permission-form.pdf) and the following text: “I request permission for the open-access journal PLOS ONE to publish XXX under the Creative Commons Attribution License (CCAL) CC BY 4.0 (http://creativecommons.org/licenses/by/4.0/). Please be aware that this license allows unrestricted use and distribution, even commercially, by third parties. Please reply and provide explicit written permission to publish XXX under a CC BY license and complete the attached form.” Please upload the completed Content Permission Form or other proof of granted permissions as an "Other" file with your submission. In the figure caption of the copyrighted figure, please include the following text: “Reprinted from [ref] under a CC BY license, with permission from [name of publisher], original copyright [original copyright year].” If you are unable to obtain permission from the original copyright holder to publish these figures under the CC BY 4.0 license or if the copyright holder’s requirements are incompatible with the CC BY 4.0 license, please either i) remove the figure or ii) supply a replacement figure that complies with the CC BY 4.0 license. Please check copyright information on all replacement figures and update the figure caption with source information. If applicable, please specify in the figure caption text when a figure is similar but not identical to the original image and is therefore for illustrative purposes only.

**R7.** We thank the Editor for pointing out this issue and apologize for the oversight regarding the inclusion of copyrighted material in Figure 1. We have removed the original Figure 1 and replaced it with a newly created figure that fully complies with the requirements of the Creative Commons Attribution License (CC BY 4.0). The revised figure does not contain any copyrighted material that would conflict with the journal's open-access policy. Furthermore, we have carefully revised the figure captions to include appropriate source information and attribution, in accordance with the journal's guidelines (see lines 148-152, p. 7 and 219-223, p. 10; Manuscript without track changes). We have also reviewed all other figures in the manuscript to confirm their compliance with CC BY 4.0 licensing requirements.

**8**. We notice that your supplementary figures are uploaded with the file type 'Figure'. Please amend the file type to 'Supporting Information'. Please ensure that each Supporting Information file has a legend listed in the manuscript after the references list.

**R8.** We thank the Editor for the comment and for carefully reviewing the figures. Now figures are part of the main manuscript, not supplementary materials, and all figure legends are complete and correctly placed in the main text as required.

**9.** If the reviewer comments include a recommendation to cite specific previously published works, please review and evaluate these publications to determine whether they are relevant and should be cited. There is no requirement to cite these works unless the editor has indicated otherwise.

**R9.** We thank the Editor for the suggestion. The reviewers did not suggest any specific additional references. However, new references have been added in the Discussion section:

Parsley KM, Daigle BJ, Sabel JL. Initial Development and Validation of the Plant Awareness Disparity Index. Nehm R, editor. CBE—Life Sci Educ. 2022;21: 64. doi:10.1187/cbe.20-12-0275

Pany P, Meier FD, Dünser B, Yanagida T, Kiehn M, Möller A. Measuring students' plant awareness: A prerequisite for effective botany education. J Biol Educ. 2024;58: 1103–1116. doi:10.1080/00219266.2022.2159491

Marmaroti P, Galanopoulou D. Pupils' Understanding of Photosynthesis: A questionnaire for the simultaneous assessment of all aspects. Int J Sci Educ. 2006;28: 383–403. doi:10.1080/09500690500277805

Fančovičová J, Prokop P. Development and initial psychometric assessment of the plant attitude questionnaire. J Sci Educ Technol. 2010;19: 415–421. doi: 10.1007/s10956-010-9207-x

**10.** Please review your reference list to ensure that it is complete and correct. If you have cited papers that have been retracted, please include the rationale for doing so in the manuscript text, or remove these references and replace them with relevant current references. Any changes to the reference list should be mentioned in the rebuttal letter that accompanies your revised manuscript. If you need to cite a retracted article, indicate the article's retracted status in the References list and also include a citation and full reference for the retraction notice.

**R10.** We have carefully checked the reference list, and we confirm that it is complete and correct. No retracted articles are cited. Additionally, new references have been added in the Discussion section (see point R9).

**Reviewers' comments:**

**Reviewer 1.**

**1.** Its a simple analysis examining whether plants disparately ignored compared to animals. The results are straight froward and clear and their analyses properly conducted with rigorous statistial analysis. The paper should be accepted as it is

**R1.** We thank the Reviewer for the positive evaluation of our manuscript and for recognizing the clarity of the results and the rigor of the statistical analyses. We appreciate the recommendation for publication.

**Reviewer 2.** The authors Silvia Guerra, Marco Roccato, Carolina Maria Oletto, Andrea Ghiani, Marco Bertamini and Luca Battaglini investigated whether PAD affacts numerosity perception and whether the difference in numerosity perception is caused by the green colour of plants or by

semantic recognition. For this, three types of experiments were conducted. The first one testing numerosity perception with coloured items, the second one with black and white items and the third one with items turned by 180° to test for semantic recognition. The study participants had two independent tasks for each experiment, the first was to estimate the number of items, the second was a comparison task. They found that PAD affects numerosity perception of plants based on semantic recognition and not on colour perception. The study is novel and investigates a phenomenon with important social impact. While the partcipant size, the experimental set-up and the statistical approach seem reasonable, I do have some doubts about the participants involved and the statistics that should be addressed in the paper.

1. Is there any information on the residence of the participants? It is suggested that people growing up in urban settings are more disconnected to nature and are more likely to have a "nature deficit". So I could imagine that they also differ in PAD or numerosity perception of plants. Did you look into this?

**R1.** We thank Reviewer 2 for raising this important issue. We agree that participants' environmental background, particularly whether they were raised in urban or rural settings, may influence their connection to nature, which in turn could affect both PAD responses and numerosity perception of plants. Unfortunately, we did not collect this information, as it was beyond the scope of the present study. However, we fully agree that including such data would enhance the interpretation of the findings. We have now acknowledged this limitation and added it as a direction for future research in the Discussion section (lines 487-492, p. 22; Manuscript without track changes).

2. Statistics: The statistical approach seems appropriate, particularly the use of mixed-models for trial-level data and binominal models for accuracy. What I miss is a reason to exclude values outside the 4-12 range - it seems a bit arbitrary to me. Furthermore, the authors don't report whether model assumptions were checked. I would like to see that the residuals are approximately normally distributed. For the binominal models, did you check for overdispersion?

**R2**. We thank the Reviewer for raising these important methodological points, which have allowed us to clarify the rationale underlying the response-range filter, to demonstrate the robustness of our findings across alternative exclusion strategies, and to explicitly report diagnostic checks for both Gaussian and binomial mixed-effects models. We address each point in detail below and in the Supplementary materials which are publicly available in the Open Science Framework (OSF) repository: https://osf.io/mzpr5/

Please note that even if the screen shows '0 bytes', the files are present in the repository. Furthermore, a brief clarification has been added to the main text (see lines 157-158, p. 7 and 269-282, pp. 12-13; Manuscript without track changes).

i) We agree that the original 4-12 response-range filter required clearer justification. Regarding the lower bound, the key distinction is between a response of 0 and responses of 1-3. A response of 0 was treated as qualitatively different, as it most likely indicates that the stimulus was not perceived at all (e.g., due to momentary inattentiveness or occasional display delays in the online experiment). In contrast, responses of 1-3 are not inherently implausible as perceptual estimates. However, they fall within the subitizing range. Regarding the upper bound, responses of 10–12 were retained following visual inspection of the response distribution, in order to preserve a symmetric buffer around the presented numerosity range (7-9). Thus, the 4–12 rule maintains a balanced window around the stimulus range while excluding values that are unlikely to reflect genuine perceptual estimates and are more plausibly due to accidental typographical entries.

**Fig. S2**. Panel figure showing typed-responses across main range-rule method

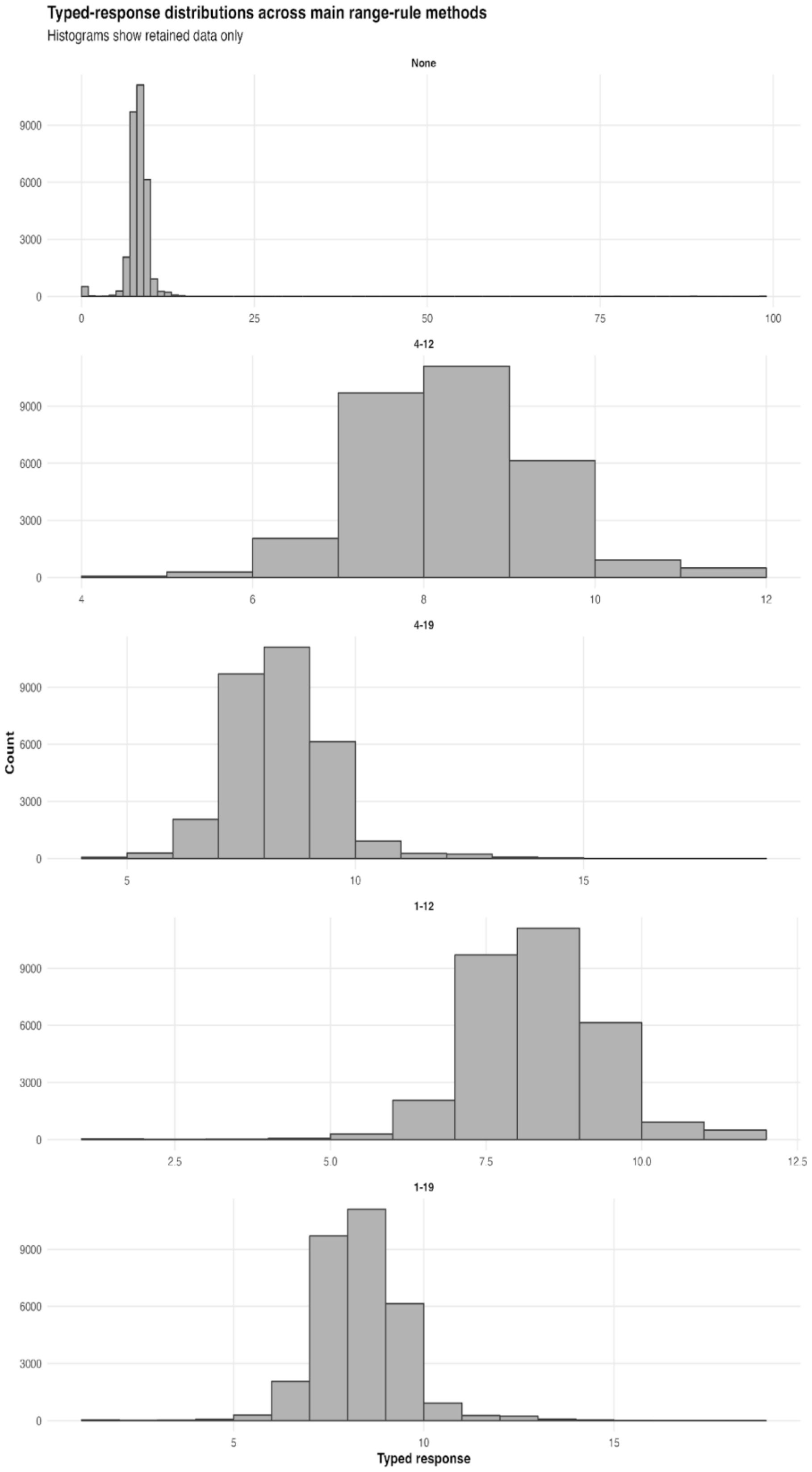

To directly address the Reviewer's concern, we conducted a comprehensive sensitivity analysis (see Supplementary Material, OSF repository) by repeating the estimation models under several alternative filtering rules: no exclusion, 4-12 (manuscript rule), 4-19, 1-12, and 1-19. The descriptive results (Table S1), model outputs (Table S2), key contrasts (Table S3), and associated figures (Figures S1–3) show that the fully unfiltered model attenuates several effects, consistent with distortion from clearly implausible responses. In contrast, across all filtered variants, the main effects of category, experiment version, and numerosity remained significant, as did the category × experiment version and experiment version × numerosity interactions. This indicates that the main PAD pattern is not driven by the specific 4-12 rule. More permissive upper bounds (e.g., 4-19) led to some attenuation of higher-order interactions, particularly category × numerosity and the three-way interaction, but the core qualitative conclusions remained unchanged. Additionally, we conducted a supplementary analysis using a participant-specific ±3 SD exclusion rule based on trial-wise estimation error. This analysis (Tables S4-6; Figure S4, Supplementary Material, OSF repository) again preserved the main category pattern and key effects, although some higher-order interactions were no longer significant. We interpret this as further evidence for the robustness of the main PAD effect, while noting that finer interaction structures are somewhat sensitive to the exclusion criterion.

**Fig. S4.** Panel showing estimation pattern across main outlier-removal methods

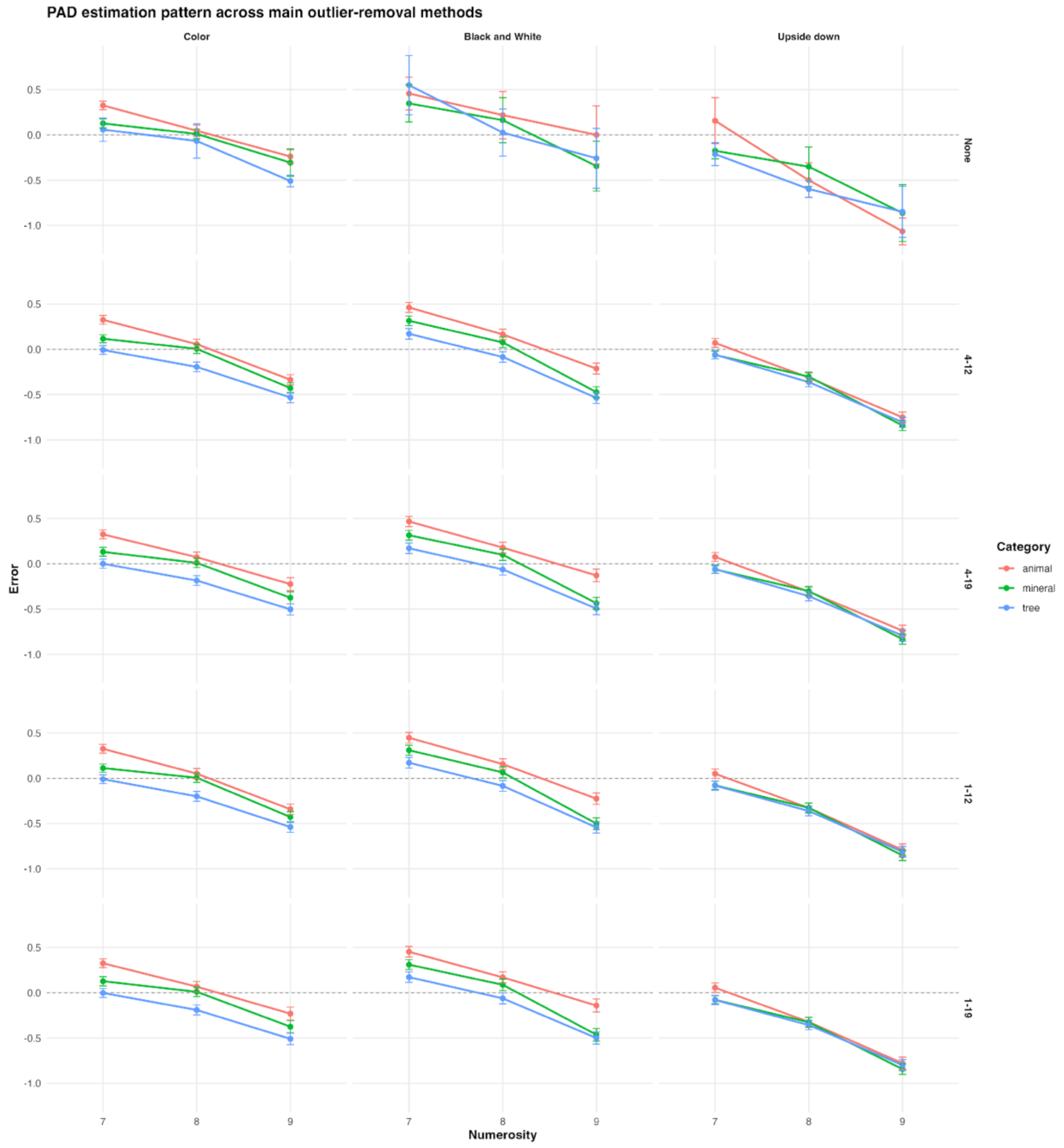


Furthermore, the same analyses were replicated using an independent software environment, specifically MATLAB R2023a, to ensure that the results were not dependent on the data processing tool employed (see Tables S7-S12 and Figure S5) The convergence of results across two independent software environments (R and MATLAB), two distinct statistical frameworks

(Wald $\chi^2$ and F-tests), and multiple outlier exclusion strategies (range-based and SD-based) provides strong evidence that the core conclusions of the manuscript are reliable, replicable, and not contingent on any specific methodological decision.

ii) We agree that model assumptions should be explicitly reported. For the linear mixed-effects model, assumptions were assessed using both visual diagnostics and formal tests. Inspection of the Q–Q plot (Figure S6, Supplementary Material, OSF repository) indicated that residuals followed the expected linear trend reasonably well in the central range.

**Fig. S6.** Visual inspection of the Q–Q plot

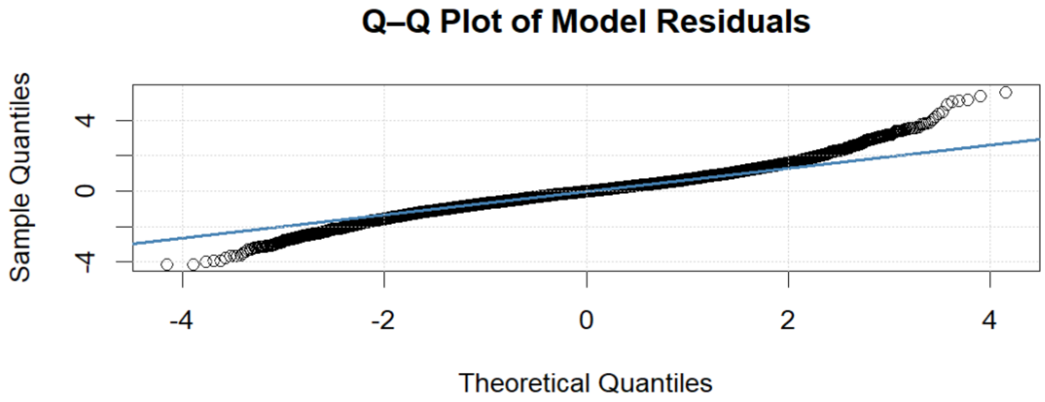


A formal test suggested deviation from exact normality (check_normality(lmm_est): $p < .001$), which is not unexpected in a dataset of this size and does not, in our view, invalidate the model. Importantly, residuals-versus-fitted plots did not indicate problematic heteroscedasticity, and the formal test confirmed acceptable variance homogeneity (check_heteroscedasticity(lmm_est): $p = .246$). Overall, while residuals are not perfectly normal, they are sufficiently close for reliable inference in a large-sample mixed-model framework. A summary of these diagnostics is reported in Table S13 (Supplementary Material, OSF repository).

iii) Finally, we agree that overdispersion should always be assessed in binomial mixed models. In both comparison models, no evidence of problematic overdispersion was found (Table S14, Supplementary Material, OSF repository).

**3.** I think the results section would benefit from some more context. It would be nice to start each section with a reminder of the leading hypothesis followed by the results.

**R3.** We thank the Reviewer for this helpful suggestion. We have revised the Results section by adding an introductory paragraph outlining the main hypotheses, in order to provide clearer context for the presentation of the results. Please refer to lines 300-313 p. 14 of the revised manuscript (Manuscript without track changes).

**4.** Figure 3: In my view the legend is not comprehensive and should include the statistical method. I would also like to see statistical differences marked with an asteriks in the figure itself.

**R4.** We thank the Reviewer for this helpful observation. We have revised the legend of Figure 3 to include a clear description of the statistical methods used. In addition, we have updated the figure to indicate statistically significant differences using asterisks. Please note that in panel A, statistical significance was difficult to clearly mark with an asterisk due to limitations in figure space. Therefore, we have chosen not to include this indication within the figure to avoid potential confusion. Please refer to the revised version of Figure 3 and its caption (lines 325-332, p. 15; Manuscript without track changes).

**5.** the resolution of the figures is poor

**R5.** We thank Reviewer for pointing this out. We have improved the resolution of all figures to ensure better clarity in the revised manuscript. Please refer to the new figures' version.

**6.** "across experiment types" vs "versions" is inconsistent wording.

**R6.** We thank the Reviewer for highlighting this inconsistency. We have revised the manuscript to ensure consistent use of terminology throughout.
